# Pharmaceutical Availability across Levels of Care: Evidence from Facility Surveys in Ghana, Kenya, and Uganda

**DOI:** 10.1371/journal.pone.0114762

**Published:** 2014-12-11

**Authors:** Samuel H. Masters, Roy Burstein, Brendan DeCenso, Kelsey Moore, Annie Haakenstad, Gloria Ikilezi, Jane Achan, Ivy Osei, Bertha Garshong, Caroline Kisia, Pamela Njuguna, Joseph Babigumira, Santosh Kumar, Michael Hanlon, Emmanuela Gakidou

**Affiliations:** 1 Department of Health Policy and Management, University of North Carolina at Chapel Hill, Chapel Hill, North Carolina, United States of America; 2 Institute for Health Metrics and Evaluation, University of Washington, Seattle, Washington, United States of America; 3 RTI International, Durham, North Carolina, United States of America; 4 Infectious Disease Research Collaboration, Kampala, Uganda; 5 Ghana Health Service, Accra, Ghana; 6 Action Africa Help International, Nairobi, Kenya; 7 Department of Global Health, University of Washington, Seattle, Washington, United States of America; 8 Department of Economics & International Business, Sam Houston State University, Huntsville, Texas, United States of America; World Health Organization, Switzerland

## Abstract

**Objective:**

In this study we use facility-level data from nationally representative surveys conducted in Ghana, Kenya, and Uganda to understand pharmaceutical availability within the three countries.

**Methods:**

In 2012, we conducted a survey to capture information on pharmaceuticals and other facility indicators from over 200 facilities in each country. We analyze data on the availability of pharmaceuticals and quantify its association with various facility-level indicators. We analyze both availability of essential medicines, as defined by the various essential medicine lists (EMLs) of each respective country, and availability of all surveyed pharmaceuticals deemed important for treatment of various high-burden diseases, including those on the EMLs.

**Results:**

We find that there is heterogeneity with respect to availability across the three countries with Ghana generally having better availability than Uganda and Kenya. To analyze the relationship between facility-level factors and pharmaceutical stock-out we use a binomial regression model. We find that the factors associated with stock-out vary by country, but across all countries both presence of a laboratory at the facility and presence of a vehicle at the facility are significantly associated with reduced stock-out.

**Conclusion:**

The results of this study highlight the poor availability of essential medicines across these three countries and suggest more needs to be done to strengthen the supply system so that stock remains uninterrupted.

## Introduction

Millions of people worldwide die or face disability each year due to diseases that have proven pharmaceutical treatments [1], [2]. In order to decrease these preventable deaths, access must increase to necessary medicines. Assuming people visit the health center when they are sick, the provision of pharmaceuticals at the health center to treat deadly disease is imperative. However, drugs are often not provided at facilities due to stock-out, or specifically, a pharmaceutical not being available due to it being out of stock. Stock-out of medicines has a profound effect on health in various ways. First, if a drug is not available then a sick patient who visits the health facility will not be able to receive the treatment they need. Patients who do not receive proper drug treatment have worse outcomes. Pasquet et al. (2010) link patients with facility-level antiretroviral medicine stock-out and find that stock-out led to higher mortality [3]. Second, if a facility experiences stock-out a patient may be less willing to visit the health facility because they do not believe they will get the care and medicine they need to be properly treated. Nabbuye-Sekandi et al. (2011) find that patients' perceptions of a health facility and their satisfaction with health services are directly linked to the availability of pharmaceuticals at that facility [4]. In addition, Hanson et al. (2005) find that availability of pharmaceuticals is a significant predictor of perceived quality of health facilities [5]. These studies imply that as availability of pharmaceuticals deceases, patients reduce their positive perception of the facility. Perceived quality of a health facility can have a significant effect on a patient's choice to utilize or not [6]. Therefore, it is important to stock medicines so people maintain confidence in the health system and continue to utilize it.

Despite the obvious need for drugs to treat patients, information on the prevalence of stock-out has been predominantly analyzed through small survey datasets and there is a dearth of information related to the determinants of stock-out. There is a large existing literature that examines stock-out and price of essential medicines using an approach developed by the WHO and Health Alliance International in 2003 [7]. The approach examines the availability and affordability of up to 50 medicines that are considered essential by the WHO. The survey has been conducted in numerous countries worldwide and is still being used today as a relevant policy undertaking [8]–[12]. Specific results from the WHO/HAI methodology are comparable across studies. Multiple studies have synthesized relevant data from numerous WHO/HAI surveys to generate medicine availability estimates [13], [14]. The WHO/HAI method has been widely used; however, it does little to take into account prevailing pharmaceutical norms within each country, such as different referral levels carrying different pharmaceuticals to address their relevant burden. In addition, another limitation of these studies is that they use surveys with small sample sizes that often lack generalizability to the entire pharmaceutical system of a country.

Other literature has looked at pharmaceutical availability at the facility level not using the WHO/HAI methodology. Buabeng et al. (2008) explore anti-malarial drug availability in Ghana and find that fewer than half of the health facilities sampled stocked the recommended first line therapy to treat malaria [15]. Despite these numerous studies looking at availability, none of them examine the facility-level determinants of availability.

There is some literature that examines the relationship between modifiable policies/factors and drug availability. Kangwana et al. (2009) investigate the effect of a nationwide pharmaceutical policy to stock artemisinin combination therapies (ACTs) in public facilities in Kenya [16]. They find uptake of the policy has been widespread; however, stock-out of artemether-lumefantrine is high, greater than 25%, two years after its introduction as a treatment for uncomplicated malaria. Davis et al. (2013) analyze availability and affordability of ACTs in five countries currently receiving Global Fund funding [17]. They find that stock-out is higher among rural facilities than urban in Ghana and Kenya and that stock-out rates were highest in rural Kenya (87% experienced at least one stock-out occurrence in the prior 3 months) and lowest in Tanzania (20.4%). Tumwine et al. (2010) analyze pharmaceutical stock-out in a single hospital in southwestern Uganda using a pre/post design. During the pre-period the hospital used a push system in which the facility was sent a package of drugs determined by the government and in the post-period it used a pull system in which the facility ordered its own drugs based on need [18]. They find that the hospital faced significantly higher stock-out while under a push system, but they acknowledge the limitations inherent in examining a single facility.

The need for increased access to pharmaceuticals drove the World Health Organization to publish its first essential drugs list in 1977, which later became the essential medicines list (EML) and is now on its 18^th^ edition [19], [20]. The EML is designed to highlight drugs that are essential to the care of diseases that plague individuals worldwide. The EML has become a ubiquitous concept with many countries adopting the strategy of creating national EMLs to treat their relative disease burdens in cost-effective ways. Ghana, Kenya, and Uganda have all developed EMLs and the pharmaceuticals included in each vary slightly depending on the country's needs [21]–[23]. The national EMLs are designed to include the pharmaceuticals that should be available to treat disease at various levels of the public health care system. For example, a referral hospital should carry specialist medicines that are necessary for care at the national level, but at the local level only a few select pharmaceuticals may be available to treat the most common diseases. Private facilities are not required to carry the essential medicines laid out by the national EML guidelines, but many do given their pertinence and cost-effectiveness.

In this study we utilize large nationally representative facility-level datasets to provide new estimates of pharmaceutical availability in three sub-Saharan African countries— Ghana, Kenya, and Uganda —and thus fill some of the existing knowledge gap on availability in the region. To our knowledge, this study is the first to examine the relationship between supply chain and facility-level factors and stock-out using nationally representative facility datasets.

## Methods

### Data

Data was collected using a standard survey instrument in each country as part of the Access, Bottlenecks, Cost and Equity (ABCE) project [24]–[26]. Field work was conducted in Kenya and Uganda from May to November of 2012 and in Ghana from June to September of 2012. A nationally representative sample of health facilities and pharmacies were visited in each country. Data on pharmaceuticals was collected from 209 facilities in Kenya, 220 facilities in Ghana and 230 facilities in Uganda. Facilities were grouped based on their ability to provide service and their designation by the various Ministries of Health in each country. We have labeled these groups of facilities as platforms and will report on them as such. Information on the facilities and platforms is summarized in Table 1. The ABCE study was reviewed and approved by the University of Washington's Institutional Review Board and the corresponding Institutional Review Boards in Ghana, Kenya, and Uganda.

**Table 1:** Pharmaceutical availability by country.

			All pharmaceuticals			Essential Medicines		
	Platform	Number of facilities	Mean number stocked out at time of survey	%	# of drugs available	Mean EML drugs stocked out	%	Total EML drugs expected
**Ghana**	Referral hospital	11	5.2	12%	44	11.5	24%	48
	Public hospital	18	2.3	6%	38	13.4	29%	46
	Health center	43	2.0	9%	22	4.7	26%	18
	Private clinic	30	2.8	10%	29			
	Community health post	65	2.4	15%	16	2.5	35%	7
	Maternity clinic	16	2.5	11%	23			
	Pharmacy	37	2.0	8%	27			
**Kenya**	Referral hospital	11	3.0	7%	41	7.6	18%	42
	District hospital	19	4.3	12%	37	10.8	26%	42
	Private hospital	17	2.9	7%	41			
	Sub-district hospital	9	6.1	17%	35	7.8	29%	27
	Health centre	54	6.6	23%	28	8.7	33%	26
	Medical clinic	18	4.0	11%	38			
	Dispensary	34	5.3	22%	24	10.3	39%	26
	Maternity clinic	20	2.6	9%	29			
	Pharmacy	16	1.9	5%	40			
	VCT center	11	0.2	5%	4			
**Uganda**	Referral hospital	13	5.5	12%	46	11.2	22%	51
	District hospital	21	3.8	9%	43	12.4	25%	50
	Private hospital	6	1.8	4%	47			
	Health center IV	39	6.8	17%	39	14.1	33%	43
	Health center III	54	6.6	21%	31	11.1	37%	30
	Medical clinic	14	2.8	8%	35			
	Health center II	46	5.7	24%	24	6.6	36%	18
	Pharmacy	37	2.7	7%	38			

Questions on specific pharmaceuticals varied slightly in each country based on country data needs, but a core group of 50 pharmaceuticals were asked about across all countries. Pharmaceuticals were included based on their necessity for treating high-burden diseases and on advice from our in-country partners. The vast majority of pharmaceuticals were from the EML of each country.

In addition to pharmaceutical data captured at the facility, various facility characteristics were asked about in the survey. Questions asked varied by country, but Table 2 summarizes the responses. Specifically, questions about the storekeeper were not asked in Ghana, whereas questions about where the facility regularly receives pharmaceuticals from were not asked in Kenya. Distances were calculated using straight line distance to the capital.

**Table 2:** Facility level characteristics by country.

	Uganda	Kenya	Ghana	Range
Order drugs and receive routine shipments	10%	19%	19%	[0,1]
Order drugs only	63%	74%	75%	[0,1]
Receive routine shipments only	26%	6%	5%	[0,1]
Receive drugs from the Ministry of Health	40%		13%	[0,1]
Receive drugs from the private market	60%		95%	[0,1]
Record keeper: admin personnel	11%	11%		[0,1]
Record keeper: medical personnel	41%	36%		[0,1]
Record keeper: pharmaceutical personnel	46%	48%		[0,1]
Record keeper: accounting personnel	10%	7%		[0,1]
Facility has a vehicle	59%	49%	67%	[0,1]
Facility has drug kits	42%	39%	0%	[0,1]
Facility has a lab	73%	84%	56%	[0,1]
Distance to the capital in decimal degrees	1.57	1.72	2.43	[0,5.7]

The dependent variable in the regressions was the number of pharmaceuticals that were stocked-out. Each drug was categorized as stocked-out if it was not available at the time of survey administration. In order for a pharmaceutical to be considered available at the time of survey administration, the drug had to be directly observed by the surveyor. General pharmaceuticals in all facilities were considered stocked-out if the facility reported normally having the drug but it was not directly observed. Essential medicines were considered stocked-out if the facility was public—thus having to adhere to the EML guidelines—and the drug was unavailable, even if they reported not typically carrying the drug. This specification creates higher drug stock-out since drugs not reported as typically carried are considered stocked-out.

### Statistical analysis

The data was structured so that each facility had a number of drugs stocked-out and a bounded number of possible drugs stocked, as well as various facility-level variables. The data structure led us to analyze the association between facility characteristics and pharmaceutical stock-out using a generalized linear model with a binomial family. Two different dependent variables were used, 1) pharmaceutical stock-out of any drug in all facilities and 2) pharmaceutical stock-out of essential medicines in public facilities. Independent variables were included where appropriate for each country. Specifically, whether or not the facility was on a push or pull system was included as a categorical variable coded as push, pull or both. We included whether or not the facility had an administrative person, an accountant, a medical personnel or a pharmacist in charge of monitoring pharmaceuticals. The presence of different types of record keepers were included as binary variables. Whether or not the facility received drug kits, whether or not the facility had a lab, and whether or not the facility had access to a vehicle were included as binary variables. Rurality of the facility was included as a categorical variable, with rural, semi-urban and urban as the three categories. Distance to the capital was coded as a continuous variable and the unit of analysis was the decimal degree. Decimal degrees were measured to the hundredth of a degree. Since all countries are at or near the equator, we found the variability in actual distance to be small enough for this to be a suitable distance measure. Whether or not a facility received pharmaceuticals from the Ministry of Health or received them from a private provider were each included as binary variables. In addition to the covariates measured at the facility level, we also controlled for platform of the facility and month that the facility survey took place—to control for potential seasonality of stock-out. Facilities were not included in the regression analysis if they reported not carrying any of the pharmaceuticals asked about in the survey. This accounted for 13 facilities (9 in Kenya, 1 in Uganda, and 3 in Ghana) when using the any drug dependent variable. No public facilities were dropped in the analysis of EML drugs since all were expected to carry the essential drugs. Regression analysis was done using the *binreg* command in Stata software version 12 and a logit link function was used to define the relationship between the independent variables and stock-out [27]. Standard errors were corrected for heteroskedasticity using White's general correction, implemented as the robust option in Stata.

## Results

### Availability of pharmaceuticals

Table 1 summarizes the stock-out variable by country and platform. Results indicate that rates of pharmaceutical stock and stock-out varied by country and platform. In general, facilities carried more pharmaceuticals the more complex the services they offered. Hospitals tended to carry over 80% of the pharmaceuticals surveyed, while community level facilities carried less than half. CHPS compounds in Ghana carried the fewest drugs on average per facility with 16 of the 54 surveyed pharmaceuticals typically available. All three countries faced similar patterns of stock-out with the low-level community public health facilities facing the highest proportion of drugs stocked-out. Pharmacies faced very low stock-out in all three countries. In addition, private medical service providers, such as private hospitals and medical clinics, faced low stock-out compared to their public counterparts.

Certain high-profile pharmaceuticals are of significant importance to address the disease burden of these sub-Saharan African countries. Table 3 summarizes stock-out of a country's first line treatment for malaria, pneumonia, and meningitis. In general, stock-out of antimalarial drugs was lower than drugs to treat pneumonia and meningitis. Across the sample, Ghana had the lowest rate of ACT stock-out with only 2% of facilities and Kenya the highest with 7%. Amoxicillin stock-out was also much lower in Ghana, at 5% compared with over 14% in Uganda and 21% in Kenya. All countries faced stock-out rates greater or equal to 10% for ceftriaxone and chloramphenicol, the drugs necessary to treat meningitis.

**Table 3:** Stock-out of essential medicines to treat malaria, pneumonia and meningitis by country.

	Uganda		Kenya		Ghana	
Drug	%	Count	%	Count	%	Count
Coartem	6%	226	7%	194	6%	204
AS+AQ					14%	199
Any ACT	6%	226	7%	194	2%	214
Amoxicillin	14%	225	21%	198	5%	205
Cotrimoxizole	4%	227	6%	195	5%	200
Erthromycin	31%	207	30%	182	15%	136
Chloramphenicol	14%	216	27%	123	10%	166
Ceftriaxone	14%	149	31%	162	10%	99

*Notes*: Percent is rate of stock-out. Count is the number of facilities that said they typically carry the drug. AS+AQ

stands for Artesunate + Amodiaquine.

### Determinants of stock-out

The results of the country specific regressions suggest that the factors influencing stock-out differ across the three countries (Table 4). Rurality of the facility did not have a significant effect on stock-out of essential medicines in any of the countries. However, in Uganda rural facilities had 59% higher odds of stock-out than urban facilities, while in Kenya rural facilities had 72% lower odds of stock-out than urban, but it was only marginally significant. In Ghana, if the facility received drugs from a private supplier they were less likely to face stock-out of any medication and essential medications. In Uganda, both facilities receiving pharmaceuticals from the MOH and from private suppliers were associated with reduced stock-out. In Uganda, presence of a vehicle was associated with lower stock-out for both any surveyed drug and essential medicines. In Ghana and Kenya the results were similar, with presence of a vehicle associated with lower stock-out; however, the effect was only significant for essential medicines. Having a lab in the facility was significantly associated with reduced essential medicine stock-out, possibly suggesting that more advanced facilities are able to test and subsequently treat only positive cases, which would drive down unnecessary use of pharmaceuticals. Where the facility received drugs from was significantly associated with stock-out.

**Table 4:** Generalized linear model results of pharmaceutical stock-out in Uganda, Kenya and Ghana.

	Uganda		Kenya		Ghana	
Dependent variable	ALL	EML	ALL	EML	ALL	EML
Both routine and order	Base	Base	Base	Base	Base	Base
Routine only	0.73	0.80	1.24	1.13	0.92	1.26
	(0.16)	(0.12)	(0.26)	(0.15)	(0.17)	(0.23)
Order only	0.65*	0.65**	0.92	0.91	2.18**	1.62
	(0.16)	(0.11)	(0.30)	(0.20)	(0.73)	(0.68)
Receive drugs from the MOH	0.75*	0.77**			0.73	1.01
	(0.13)	(0.09)			(0.19)	(0.16)
Receive drugs from private	0.71	0.75**			0.50***	0.34***
	(0.17)	(0.10)			(0.13)	(0.12)
Record keeper-admin	0.57	0.49*	0.78	1.10		
	(0.23)	(0.19)	(0.28)	(0.38)		
Record keeper-medical	0.85	0.97	0.54	0.76		
	(0.25)	(0.25)	(0.21)	(0.29)		
Record keeper-pharmacist	0.76	0.85	0.61	0.89		
	(0.25)	(0.22)	(0.22)	(0.34)		
Record keeper-accountant	0.98	0.83	0.75	1.05		
	(0.37)	(0.31)	(0.25)	(0.53)		
Presence of a vehicle	0.74*	0.81*	0.75	0.68***	0.98	0.80
	(0.13)	(0.10)	(0.14)	(0.09)	(0.20)	(0.14)
Facility receives drug kits	1.20	1.02	1.04	0.86		
	(0.17)	(0.10)	(0.22)	(0.12)		
Presence of a lab	0.81	0.73**	0.76	0.57***	0.84	0.72**
	(0.18)	(0.10)	(0.27)	(0.11)	(0.17)	(0.10)
Urban	Reference	Reference	Reference	Reference	Reference	Reference
Semi-urban	1.09	0.94	1.04	0.87	1.24	1.22
	(0.19)	(0.12)	(0.21)	(0.12)	(0.32)	(0.21)
Rural	1.59**	1.07	0.58*	1.15	0.71	1.08
	(0.31)	(0.17)	(0.17)	(0.20)	(0.15)	(0.22)
Distance to capitol	1.07	1.00	1.01	1.12**	1.09*	1.03
	(0.12)	(0.08)	(0.07)	(0.05)	(0.05)	(0.04)
N	227	150	186	114	217	137

*Notes*: Coefficients are odds ratios. Robust standard errors are included in parentheses. Dependent variable ALL implies regression was run with all drugs included in the regression model. Dependent variable EML implies the regression was only run on public facilities and only on EML drugs. *,**, and *** denote significance at the 0.1,0.05, and 0.01 level respectively. Regressions also controlled for facility type and the month of survey but results are not shown.

## Discussion

In this analysis of over 600 health facilities in Ghana, Kenya, and Uganda we have shown that pharmaceutical stock-out is still a persistent problem despite large efforts in each country to strengthen the provision of pharmaceuticals. Availability of essential medicines was strikingly low, with over 30% of those expected to be available not at the facility. Even if availability is low, it is important to note that facility specific stock-out does not necessarily imply that a patient does not receive pharmaceuticals. A patient could go to a private pharmacy or another provider to get medication. However, this entails multiple trips by the patient and as the number of required trips increases, the likelihood a patient will visit the subsequent facility decreases, thus decreasing their probability of getting the pharmaceuticals they need. This has the potential to have a profound impact on the health of these populations if patients are not receiving the pharmaceuticals they need. It is necessary that facilities carry the pharmaceuticals expected of them so that patients get the care they need and expect.

Regression analysis suggests the presence of vehicles at the facility is associated with reduced stock-out. One potential way to alleviate stock-out in Ghana, Kenya, and Uganda is to provide vehicles to facilities so they can seek out medications when their stock is low. This intervention would give the facility more autonomy and accountability in dealing with pharmaceutical procurement. Despite our *a priori* hypothesis that presence of pharmacists would reduce stock-outs, we found no evidence that this was the case. Furthermore we found that there were no significant differences in stock-out based on who was in charge of the pharmaceutical records. This finding implies that facilities without experienced pharmaceutical personnel may still be capable of monitoring their own stock. Given this outcome, supplying vehicles to facilities, even those without a pharmacist, would be a useful intervention.

The results from our analysis on availability were consistent with those found in the existing literature. Robertson et al. (2009) found the rate of pharmaceutical availability in our surveyed countries to be lowest in Ugandan district hospitals and primary health care centers, each at 29% [28]. We found availability to be much higher, which could be due to the Robertson study using specific formulations of the drug, rather than asking about the drug regardless of formulation, as we did. In addition, the Robertson study uses a standard 20 medicines across 14 different African countries and did not incorporate whether Uganda utilizes those 20 medicines. Davis et al. (2013) find stock-out rates of ACTs among our surveyed countries to be highest in Kenya and lowest in Ghana [17]. Although their stock-out measure is not directly comparable because it incorporates any stock-out within the past 3 months rather than at the time of survey, their results seem to show a similar trend. Our analysis provides more robust results because we analyzed more drugs and used directly observed availability rather than self-reported availability.

This analysis faced three specific limitations. First, in our survey we did not ask about availability of specific formulations of a pharmaceutical. If we had information on this we would be better able to assess the importance of some drugs over others. For example, a facility carrying an antibiotic for use with pediatric patients would still be considered not having stocked-out of that antibiotic even if the antibiotic for adults was stocked-out. However, the likelihood that this limitation would have any real effect on the analysis is minimal since it is likely that facilities stock pharmaceutical formulations that are prescribed to them by the EML and not others. Second, we did not measure if the drug was effective. Counterfeit drugs are known to exist in Africa but very little is known about their prevalence due to lack of survey data [29], [30]. We attempted to increase the probability of correctly classifying drugs by ensuring that our trained RAs directly observed the pharmaceuticals during survey administration. Future analysis should focus on determining, in addition to if a facility stocks medicines, if the medicines they stock are effective and safe. Third, we did not have outcomes at the patient level and therefore were unable to assess the relationship between pharmaceutical stock-out and patient outcomes. Although it is not exactly relevant as a limitation of the analysis presented in this study, it is an area we would like to explore with more rich datasets that link facility pharmaceutical availability with patient outcomes data.

## Conclusion

Very little existing literature has explored the relationship between facility-level determinants and availability of pharmaceuticals. Future research should attempt to better understand the causal mechanisms that drive pharmaceutical procurement and subsequent stock-out. In addition, countries should take a close look at their own health systems to understand where the need exists for better pharmaceutical monitoring to improve drug availability.

In conclusion, Ghana, Kenya, and Uganda still have a long road ahead before necessary essential medicines are available in the health facilities as they are mandated. Investment should be made to strengthen the monitoring system of pharmaceutical procurement and more autonomy should be given to facilities to monitor their stock. With increased availability of essential medicines the health of these countries should improve.

## Supporting Information

S1 Data **Study data.** This supporting information file contains the data used in this study.
(DTA)
